# The role of institutional quality in the international trade of a Latin American country: evidence from Colombian export performance

**DOI:** 10.1186/s40008-021-00253-5

**Published:** 2021-11-19

**Authors:** Carlos Abreo, Ricardo Bustillo, Carlos Rodriguez

**Affiliations:** University of the Basque Country, Lehendakari Aguirre, 83, 48015 Bilbao, Spain

**Keywords:** Institutional quality, Free trade agreements, Trade, Exports, Trade balance, Trade gravity model

## Abstract

This paper analyses the relevance of Colombian institutional quality in recent years in terms of the performance of its exports within a framework of trade openness. Based on the trade gravity model, we examine the effect of governance on the evolution of Colombian exports through an econometric approach that identifies, on the one hand, the influence of institutional quality, and on the other hand, the influence of the institutional distance between Colombia and its trading partners. We use a panel data set for 2005–2018, through which the export flows from Colombia to 136 of its trading partners are considered. The findings indicate that Colombian institutional quality and the institutional distance between the country and its partners are statistically significant and affect its foreign sales. Similarly, there is a prominent influence of regulatory quality and the rule of law variables in the performance of Colombian exports in relation to other variables included in the model. We conclude that the Colombian government must improve its institutional quality considerably as a fundamental step towards boosting its overseas sales, not least because the country’s institutional distance from the world average is notable, which also affects its exports.

## Introduction

In recent years, relevant research has explored the effect of institutional variables, which have received a great deal of attention (Anderson and Marcouiller 2002; Dollar and Kraay 2004; Levchenko 2007; Egger et al. 2011; Egger and Nigai 2015; Álvarez et al. 2018), and it is widely understood that higher institutional quality and a better atmosphere of governance reduce business costs and promote an efficient business environment, which can improve bilateral trade flow between nations (Wu et al. 2012).

Colombia, as one of the countries experiencing one of the highest economic growth rates in Latin America over the past decades, has developed a dynamic policy of trade openness in order to promote its economic development through the promotion of its exports. Nonetheless, although the trade openness policy has been defined by different studies as a successful policy to improve bilateral trade, this has been widely questioned in Colombia. The nation’s trade balance, which has historically been in deficit, has deepened further in recent years, presenting one of the largest imbalances in Latin America after only Mexico and Argentina (ECLAC 2020). Based on this negative trend established within a context of complex socioeconomic factors, such as high rates of informality, greater concentration of wealth, controversial electoral processes, drug trafficking and internal armed conflict, among others related to governance, this study attempts to evaluate the effect of Colombian institutional quality on its export trade flows. The results will allow us to simultaneously infer the extent to which the quality of Colombian governance affects its exports, and to present the findings of a study that could be replicated in similar Latin American countries. Consequently, the findings of the study will allow us to offer some policy suggestions to address overcoming the deficiencies related to governance in Colombia.

This paper contributes to previous research into the influence of governance on international trade, with a focus on emerging economies that have followed the path of trade liberalisation promoted by developed economies. This study is the first to address the effect of institutional quality on exports from a country like Colombia, which has been deeply affected by a long internal armed conflict that has affected the numerous efforts made by national authorities over recent decades to improve their governance indices. We apply the trade gravity model to a cross-sectional dataset of bilateral exports between Colombia and 136 countries from 2005 to 2018, before we then use a group of institutional quality indices elaborated by the World Bank (WB) to test the influence on Colombian exports, firstly, from the institutions, and, secondly, from the institutional distance between Colombia and its trading partners. Furthermore, we include variables based on the new trade theory framework to assess the endogenous characteristics of the exporting country and the effect of them on trade flows. Finally, the econometric model is estimated by the Poisson pseudo-maximum likelihood (PPML) approach (Silva and Teneyro 2006), which is the most suitable econometric procedure in the presence of a large number of zeros, potential endogeneity, and econometric drawbacks.

This paper is organised as follows. The first part describes the influence of institutions on international trade, as well as the performance of Colombian exports and the country’s institutional quality over recent decades. The second part of this paper will present the methodological approach, specification, and research data. Finally, it will offer its findings, focussing on the discussion and central conclusions.

## Influence of institutions on international trade

The role of institutions in society has been widely analysed by leading authors. For Schmoller (1900), institutions are habits, rules of morality, customs and laws that, when related to each other, establish a system. Commons (1934) defines institutions as collective acts that control individual acts, suggesting that society is made up of individual institutions such as the state, the family, the corporation, and the commercial association among others. Moreover, Hayek (1967) proposes that institutions are rules that conduct society, which can be divided into two classes. The first class is called “organisation” and involves simple rules which do not evolve, have defined objectives, and have been deliberately created. The second class is “spontaneous rules”: complex rules with no defined objectives which are constantly evolving and have been created in an unplanned or unconscious way. Furthermore, North (1990) divides institutions into formal constraints (such as rules, laws, and constitutions) and informal constraints (such as standards of behaviour, conventions, and self-imposed codes of conduct), claiming that formal institutions can change rapidly while informal rules cannot. In particular, the influence of institutions on the economy has been addressed by authors such as Acemoglu et al. (2005), who place institutions as key to economic success, dividing them into extractive and inclusive categories. Moreover, they claim that extractive institutions favour the elites and restrict long-term growth while inclusive institutions are linked to political democracy and promote economic growth. Finally, Chavance (2008) supports the relationship between institutional quality and economic growth, establishing that good institutions are based on economic freedom, rule of law, private property, flexible labour markets, clearly delineated property rights, and shareholder-oriented corporate governance.

Furthermore, the effects of higher institutional quality on international trade have been highlighted by trade literature over the last decades, supporting the idea that better institutions and governments will increase international trade flow (Bilgin et al. 2018). This hypothesis is strongly supported by Anderson and Marcouiller (2002), who argue that bilateral trade is positively affected by the quality of the institutions involved. In the same vein, Álvarez et al. (2018) state that institutional quality increases bilateral trade and this effect increases over time. Furthermore, Linders et al. (2005) conclude that the quality levels of institutions in both the exporting and importing countries increase the volume of trade between them. Likewise, Li and Samsell (2009) find that countries with a reputation for strong governance have a higher trade volume than those without. This view is supported by Jalilian et al. (2007), who point out that institutional development reduces information imperfections, increases economic incentives, and reduces transaction costs. Similarly, Chowdhury and Audretsch (2014) state that higher institutional quality and good governance reduce trade costs and the risks of defaulting while Yu et al. (2015) go further, claiming that better institutions, both formal and informal, ease trade. These statements have been proven by researchers who found that the level of institutional quality has a statistically significant positive influence on trade (de Groot et al. 2005). The evidence reviewed indicates a positive and relevant connection between institutions and trade.

The significance of the connection between institutions and trade is reflected in existing research that affirms how weak institutions can restrict international trade with a negative effect similar to that of tariffs (Álvarez et al. 2018; Anderson and Marcouiller 2002). Moreover, poor institutional quality can obstruct trade and lead to poor performance in manufactured exports (Méon and Sekkat 2004). Francois and Manchin (2013) affirm that the influence of institutions on trade is most notable in low-income countries. This statement suggests that carrying out public reforms to improve institutional quality should be a critical factor in government policy in order to obtain greater bilateral exchanges, particularly in developing nations. However, Kaufmann et al. (2011) find crucial differences between the achievements in institutional quality within developed and emerging economies, with the former demonstrating more improvements in governance.

The remarkable role of institutional quality in promoting trade, based as it is on strong and reliable institutions in both exporting and importing countries, is also explained by the similarity in the quality of their institutions. In this regard, Kostova (1997) introduces the concept of institutional distance, defined as the difference between the institutional profiles in the original and host countries. Since this idea was introduced, institutional distance has gained importance in international trade studies (Beugelsdijk et al. 2018). Some researchers who have analysed the difference in institutional quality between nations have found that most bilateral trade takes place between economies with high standards of institutional quality, in particular when the difference between their indicators is small (Álvarez et al. 2018). The importance of the similarity in institutional quality is also supported by de Groot et al. (2005), who state that countries with similar quality of governance trade more with each other, showing that higher differences in quality of governance between exporting and importing countries limit bilateral trade flow. However, there are concerns regarding the rigour during the construction of the indicator and the subsequent comparability of its results with similar research (Bae and Salomon 2010). Nonetheless, Kostova et al. (2019) point out that the richness of the institutional perspective provides several opportunities to analyse its cross-border influence on numerous strategic and organisational outcomes.

Furthermore, there is a growing body of research that recognises the importance of individual institutional variables in the economic success of nations. One of the most critical of these variables is the rule of law. As noted by Gani and Scrimgeour (2016), the rule of law is based on the interaction between citizens and institutions, and the strength of the law can be essential in promoting investment and boosting economic performance. This variable is considered so noteworthy that some studies measure the institutional quality of nations directly through it (Dollar and Kraay 2004). Its importance is also reinforced by Zeynalov (2017), who claims that the rule of law is one of the critical factors for improving bilateral trade amongst nations. Likewise, Álvarez et al. (2018) identify in their empirical research that the rule of law demonstrates one of the strongest connections with trade volumes. Finally, Gani and Scrimgeour (2016) point out that the defence of the rule of law, effective enforcement of business and trade agreements, absence of bureaucracy, an improved regulatory atmosphere, and the freedom of citizens to exercise their political and civil rights are vital aspects to promoting trade.

The risk of default or risk of no-payment generates uncertainty in trade integration, and this risk is defined through contract enforcement mechanisms. In the 1960s, Olson (1996) addressed the issue and claimed that undeveloped and inefficient institutions, such as weak enforcement mechanisms, could limit productive cooperation and thereby affect trade. Similarly, Ranjan and Lee (2007) demonstrate that imperfections in the enforcement of contracts can reduce the volume of trade in both differentiated and homogeneous goods. Nunn (2007) goes further regarding the significance of this factor in bilateral trade, claiming that application of the contract has a greater effect on the international pattern of trade than the country’s capital endowments and skilled labour combined. However, although conventional trade theory states that the endowments of a country, its level of technology and its competitiveness explain trade, Gani and Scrimgeour (2016) claim that poor governance can create limitations to trade, making the vital role of good institutions in economic progress yet more evident.

Another major factor that directly affects bilateral trade flows is corruption. On this matter, Krueger (1974) points out that trade openness positively affects competitiveness and consequently reduces the effects of corruption whereas trade barriers create bureaucracy in business processes and, as a consequence, illegal benefits. Additionally, Anderson and Marcouiller (2002) state that corruption and weak legal enforcement of contracts increase the risks to trade with countries exhibiting such weaknesses harming trade flow. Similarly, Nunn (2007) finds that the quality level of democracy is positively linked to bilateral trade flow, and this view is supported by Yu (2010), who affirms that a higher level of democracy improves bilateral trade exchange. In their study of the Asia–Pacific region, Helble et al. (2009) analyse the influence of institutional transparency on trade, finding that greater transparency of the trade environment through greater predictability and simplification of procedures has a substantial impact on trade costs.

To determine the effects of institutional quality on bilateral trade, some authors have gone beyond the analysis of formal institutional factors. Li and Samsell (2009) claim that previous studies of institutional quality mainly consider formal institutions and disregard informal ones, such as the governance environment. According to Yu et al. (2015), trust can moderate the risk of default assumed by exporters in an environment of malfunction of formal institutions, and consequently improve economic results. This view is supported by Guiso et al. (2009), who claim that low levels of trust can negatively affect bilateral trade. Furthermore, institutional quality can indirectly affect bilateral trade due to the discouragement of foreign direct investment (FDI), taking into account that this factor is a determinant of international trade (Mauro 1995). This statement suggests that the expected positive effect on trade through the attraction of FDI would not be achieved with weak government institutions (Jude and Levieuge 2017). Finally, Demir and Hu (2016) also state that institutional differences can create an entry barrier for FDI, consequently hampering trade.

## Colombian export performance and institutional quality

Since the early 1990s, following international trends, Colombia has developed a trade policy that promotes its integration with international markets, seeking a positive impact on its economic growth. To do this, former President César Gaviria Trujillo passed an unprecedented set of reforms in 1991 which aimed to encourage what he called an *Apertura Comercial* process. As noted by García et al. (2014), Gaviria’s aim of opening up the economic system was to encourage a more productive and efficient national economy. However, to date, there is little agreement on the commercial success of this process due to the few, inefficient measures developed by different governments to support and promote the competitiveness and productivity of national companies in a constantly changing economic scenario. Additionally, it is important to mention that Colombia began to sign its most important bilateral trade agreements from 2009. As confirmation of this statement, Colombia strengthened its trade relations through FTAs with the European Free Trade Association (EFTA) in 2009, Canada in 2011, the United States (US) in 2012, the European Union (EU) in 2013 and, more recently, with the countries of the Pacific Alliance (PA) in 2015 and the Republic of Korea in 2016 (MinCIT 2019). In this sense, it is essential to clarify that the trade agreements most prominent in Colombia are those signed with the US and the EU, as these agreements represented more than half of Colombian trade between 2005 and 2018, despite the fact they have contributed to deepening the deficit in Colombia's trade balance in recent years (MinCIT 2018a).

**Fig. 1:**
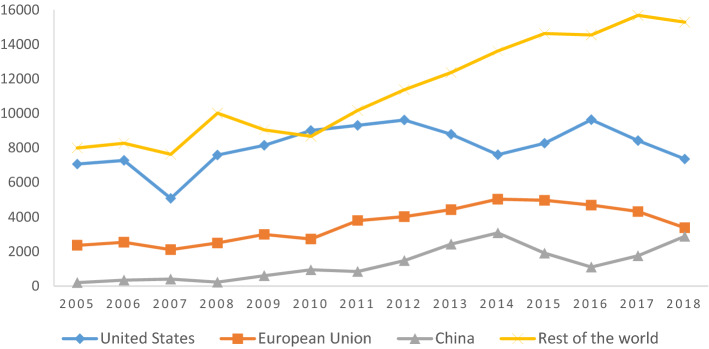
Colombian exports by destination in constant millions of USD (base year = 2000). Source: Own elaboration based on DANE (2020a). Deflated values based on the Export Price Index from BanRep (2020)

Figure 1 shows the performance of Colombian exports by destination at constant prices (base year = 2000) from 2005 to 2018.

The information displayed in Fig. 1 can be analysed in different ways. On the one hand, it suggests that the US is the main destination of Colombian exports. The US hegemony as the leading destination of Colombian exports has reduced in recent years, especially after the FTA between the parties came into force. Similarly, Colombian exports to EU member states have experienced a notable reduction after the FTA between the parties came into effect. Moreover, China has emerged as one of the most prominent destinations for Colombian exports.

It is important to note that Colombian exports are mainly composed of oil and mining goods. According to MinCIT (2018b), this sector contributed 63.3% of national exports, growing by 17.5% in 2018 on 2017. More precisely, 59.1% of Colombia’s total exports in 2018 were made up of oil and its derivatives, with that sector growing by 6.5% compared to 2017, reflecting its strategic importance for the economy. In this regard, Karabulut et al. (2020) examine the effect of economic uncertainty on international commodity trade, highlighting the negative effect of trade wars on the exchange of this type of goods, which represents a serious risk for Colombian commodity exports, given their relevance in the current scenario of economic uncertainty. Furthermore, as noted by Zeynalov (2017), volatility in international oil prices has harmed globalisation in oil-producing countries, which have generally used the revenues from this industry ineffectively. Moreover, Haddad et al. (2013) state that diversification of exports allows a country to manage the risk generated by the volatility transmitted by trade, thereby obtaining the benefits of openness. Colombia faces a significant challenge in this regard. A reduction of its high dependence on the oil industry in terms of exports, and an improvement of its institutional quality might both drive the diversification of exports. Consequently, Moenius and Berkowitz (2011) claim that countries with lower institutional quality that carry out institutional reforms can experience advances in the diversity of their exports. In view of that, more substantial and appropriate efforts are required to ensure the diversification of Colombian exports and thus avoid an exchange crisis resulting from a very probable depreciation of the Colombian peso due to the expected reduction of its oil exports in the coming years (Presidencia de la Republica de Colombia 2020). Regarding this probable state of affairs, Bahmani-Oskooee et al. (2019) recognise the negative effect of the devaluation of the local currency on Kazakhstan’s exports, which, like Colombia, is an economy highly dependent on oil sales. Nonetheless, Bahmani-Oskooee et al. (2020), in their study on the effect of the exchange rate on sectoral trade between a developing country such as Pakistan and the US found that in a situation of rupee depreciation, the trade balances of some sectors may benefit (vegetables, fresh or dried; manufactured leather; and footwear, among others), which shows that the probable depreciation of the Colombian peso could also have a positive influence on certain sectors, if the Colombian economy and, eventually, its trade patterns, were adequately diversified. Therefore, it is essential to point out that, on the one hand, the Colombian authorities must focus their short-term economic policies on avoiding the depreciation of the Colombian peso and harming its exports, while on the other hand, they must focus their long-term policies on ensuring adequate diversification of their trade pattern in order to avoid significant damage to the economy. Similarly, and as stated by Gupta et al. (2019), Colombian policymakers should also offer incentives to exporters (e.g., insurance coverages, low-interest rate loans, tax incentives) to maintain international trade in times of economic uncertainty caused principally by geopolitical risks.

**Fig. 2:**
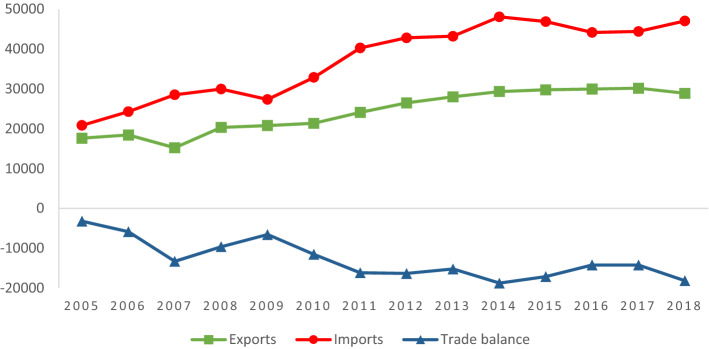
Colombian trade balance in constant millions of USD (base year = 2000). Source: own elaboration based on DANE (2020b). Deflated values based on the Export Price Index and Import Price Index from BanRep (2020)

Figure 2 shows the Colombian trade balance performance at constant prices (base year = 2000) from 2005 to 2018.

Figure 2 reflects the performance of the Colombian balance of trade, which shows a clear deepening of its trade deficit. Although exports show a slight upward trend in the period analysed, the upward trend of imports is much higher. This situation has generated an increase in the Colombian trade deficit. During the period analysed, deepened with greater intensity from 2009 and reaching its highest level in 2018. However, as mentioned above, the trade deficit has continued since 2005, intensifying after the signing of various FTAs by the Colombian government as a key policy for trade integration in global markets. Regarding imports, DANE (2019) states that the main suppliers exporting to Colombia are the US, China and the EU, respectively. It is important to note that China went from being a modest supplier of products to Colombia in 2005, to the second largest source of Colombian imports in 2018, partly to the detriment of imports from the US and the EU.

Furthermore, as noted by several studies, there is a strong relationship between export performance and the institutional quality of nations. Regarding Colombia, the effects of good governance on exports remains unclear. According to Franz (2019), Colombia accurately exemplifies the inadequacies of good governance in terms of institutions and economic development, suggesting that Colombian institutional quality is not a factor that encourages national exports.

In this regard, this study aims to determine the extent to which Colombian institutional quality influences its export performance. The institutional quality of the countries involved in the study are based on figures taken from the World Bank’s Global Governance Indicators (WGI) prepared by Kaufmann et al. (2011) and is shown in Table 1.

**Table 1:** Definition of World Bank governance indicators (WGI). Source: Kaufmann et al. (2011)

Indicator	Definition
Control of corruption	The extent to which public power is used for private gain, counting on small and large forms of corruption, as well as the management of the State by elites and private interests
Government effectiveness	The quality of public services, the capacity of the public function and its independence from political pressures; and the quality of policy formulation
Political stability and absence of violence/terrorism	The probability that the government will be damaged by unconstitutional or violent affairs, including terrorism
Regulatory quality	The government's ability to provide strong policies and regulations that enable and promote the development of the private sector
Rule of law	The extent to which agents trust and accept the rules of society, including the quality of contract enforcement and property rights, the police, and the courts, as well as the probability of crime and violence
Voice and accountability	The extent to which citizens participate in the selection of their government, freedom of expression, freedom of association and freedom of the press

The six WGIs comprise broad dimensions of governance and allow us to determine the specific effects of each indicator on Colombian exports. Moreover, the WGIs are considered the most detailed and geographically complete set of institutional indices currently available (Álvarez et al. 2018).

**Fig. 3:**
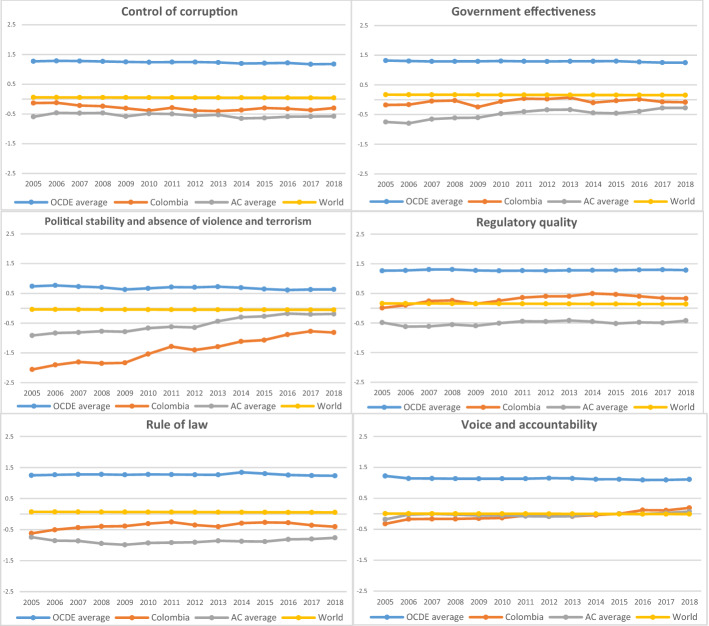
Colombian governance indicators compared with OECD and AC countries, and the world average. Source: The World Bank (2020)

Figure 3 illustrates Colombian governance indicators compared to the average among the Organisation for Economic Co-operation and Development (OECD) countries, the Andean Community (AC) countries and the whole world from 2005 to 2018.

Figure 3 illustrates the remarkable institutional distance across all the governance indicators between Colombia and the OECD countries between 2005 and 2018; however, the institutional distance is more noticeable in indicators such as control of corruption, government effectiveness, and rule of law. Regarding the institutional distance between Colombia and the AC countries, the former performs better across the majority of indicators (control of corruption, government effectiveness, regulatory quality, and rule of law). By contrast, the AC countries demonstrate a higher level of both political stability and the absence of violence and terrorism, as should be expected following the long period of internal conflict that Colombia has experienced. However, it is essential to highlight the upward trend in this indicator since 2012, when the government of the former President Juan Manuel Santos and the *Fuerzas Armadas Revolucionarias de Colombia* (FARC) began formal negotiations to end the internal armed conflict that has lasted more than 50 years (Lopez et al. 2019). Moreover, the Colombian index performance reflects a higher upward trend in the voice and accountability indicator since 2013. Finally, Fig. 3 also shows the evolution of the global average institutional indices, most of which are higher than the Colombian indices (except in regulatory quality, and voice and accountability, in which a similar evolution is observed). This last comparison allows us to infer that the quality level of Colombian governance is lower still than the global average, which indicates, on the one hand, a wide margin for institutional improvement and, on the other, the poor institutional quality of the country.

According to the empirical findings obtained by different authors about the effect on trade of institutional quality, it is important to remark on the need to improve the quality of Colombian institutions in order to positively influence exports. The OECD (2013) highlights a notable change in Colombia owing to the implementation of public policies in the last 10 years that seek to strengthen its institutional quality and to promote sustainable and inclusive economic growth. However, as evidenced in Fig. 3, Colombian institutional quality is still some distance from the average level of OECD countries, which are characterised by high standards of governance.

## Methodological approach, estimation and data

In light of this discussion, this study implements the trade gravity model to determine the effects of institutional quality on Colombian exports. This methodology is a successful approach to analysing the performance of bilateral trade flows based on variables such as distance, the size of the country’s economy, and other factors that could facilitate, or impede, trade. This approach allows us to test what recent studies have indicated regarding the influence of institutional variables on bilateral trade.

The trade gravity model was first introduced by Tinbergen (1962) and Poyhonen (1963), who formulated this method based on the *Law of Universal Gravity* formulated by the English physicist Isaac Newton. The approach states that the economic forces of trade can explain bilateral trade between origin and destination, and where a series of variables converge to promote or restrict trade flow between nations (Bergstrand, 1985).

According to Anderson and van Wincoop (2003), the trade gravity equation is modelled as follows:

$$X_{ij} = \alpha_{o} Y_{i}^{ \propto 1} Y_{j}^{ \propto 2} D_{ij}^{ \propto 3} Z_{ij}^{ \propto 3} n_{ij}$$

The equation establishes the relationship between a country’s exports ($X_{ij}$), the income levels of the countries of origin and destination ($Y_{i}^{ \propto 1}$; $Y_{j}^{ \propto 2}$), the physical distance between them ($D_{ij}^{ \propto 3}$), and all the factors that might impede trade ($Z_{ij}^{ \propto 3}$).

This empirical study considers Colombian exports to the 136 partners that constitute 99% of its export trade. The analysis begins in 2005, the year in which Colombia’s trade openness intensified, and ends in 2018, to avoid the impact of COVID-19 on the institutional quality of the nation. The variables implemented in the model vary according to economic, historical, geographical, and trade integration features.

Our specification follows the proposal of Álvarez et al. (2018), which estimates the effect of institutional barriers on exports based on the new theory of trade (NTT), wherein assumptions such as love for variety preferences, increasing returns to scale technologies, and iceberg transport are considered. Therefore, their proposal considers the influence of transport and non-transport factors as linked to trade cost. With this in mind, we include other variables connected to the context of Colombian trade, and we determine whether institutional variables affect Colombia’s bilateral trade with its main partners.

Table 2 exhibits the variables implemented in the model proposed.

**Table 2:** Information of variables implemented in the model. Source: Own elaboration

Variable	Description	Update date	Source	Expected sign
X_*Colj*_	Exports from Colombia to its partners in constant USD	June 19, 2020	World Integrated Trade Solution	
LogDIST_*Colj*_	Log Distance in kilometres between country *i* to country *j*	March 30, 2019	CEPII	−
CONTIG_*Colj*_	Common physical border between country *i* and country *j*	March 30, 2019	CEPII	+
COMLANG_*Colj*_	Origin and destination share a common legal language that is spoken by at least 9% of the population of both countries	March 30, 2019	CEPII	+
LogGDP_*j*_	Log Gross domestic product of destination country in constant USD	July 30, 2019	World Bank Data	+
LogLABCOMP_*Col*_	Log labour competitiveness of origin	August 1, 2020	Calculated by authors with data from the United Nations	+
WTO_*j*_	Colombian partner is a member of the WTO	March 30, 2019	CEPII	+
OECD_*j*_	Colombian partner is a member of the OECD	June 30, 2020	OECD	+
FTA_*Colj*_	Origin and destination country with Trade Agreement in force	May 8, 2019	WTO	+
Control of corruption_*Col*_	Colombian control of corruption: estimate	June 30, 2020	World Bank Data	+
Control of corruption_*j*_	Colombian partner’s control of corruption: Estimate	June 30, 2020	World Bank Data	+
Government effectiveness_*Col*_	Colombian government’s effectiveness: estimate	June 30, 2020	World Bank Data	+
Government effectiveness_*j*_	Colombian partner’s government’s effectiveness: estimate	June 30, 2020	World Bank Data	+
Political stability_*Col*_	Colombian political stability: estimate	June 30, 2020	World Bank Data	+
Political stability_*j*_	Colombian partner’s political stability: estimate	June 30, 2020	World Bank Data	+
Regulatory quality_*Col*_	Colombian regulatory quality: estimate	June 30, 2020	World Bank Data	+
Regulatory quality_*j*_	Colombian partner’s regulatory quality: estimate	June 30, 2020	World Bank Data	+
Rule of law_*Col*_	Colombian rule of law: estimate	June 30, 2020	World Bank Data	+
Rule of law_*j*_	Colombian partner’s rule of law: estimate	June 30, 2020	World Bank Data	+
Voice and accountability_*Col*_	Colombian voice and accountability: estimate	June 30, 2020	World Bank Data	+
Voice and accountability_*j*_	Colombian partner’s voice and accountability: estimate	June 30, 2020	World Bank Data	+

Table 2 exhibits the standard variables implemented in the trade gravity model as control variables such as contiguity and common language. Physical distance is included as a proxy for transport cost. Additionally, the GDP of the destination is included as a variable that measures the economic dimensions of the country’s partners. The variable LABCOMP depends on productivity and wages, and is established via the link between GDP and the number of workers in a country. According to Álvarez et al. (2018), its incorporation into the model is based on the so-called new trade theory (NTT), and more precisely, on the Krugman–Dixit–Stiglitz model. This variable suggests that the higher labour productivity is, the lower the marginal labour requirements become, and, therefore, wages can have a positive correlation with productivity. Moreover, the model incorporates variables that reflect important trade integration mechanisms aligned with the Colombian strategy of trade openness, such as FTAs signed between the parties and whether its partner is a member of the WTO and the OECD.

In addition to the variables mentioned above, Table 2 displays six governance indicators. The World Bank (2020) provides the governance indicators implemented in the model, and they reflect each dimension of the institutional quality of a country. The score for each index ranges from − 2.5 to 2.5, with the latter denoting higher quality. Furthermore, it is essential to note that we lag the institutional variables in our specifications because we consider that they have a lagged effect on trade (Gani and Scrimgeour 2016), and also to avoid endogeneity problems.

Table 3 shows the descriptive statistics of the variables used in the empirical study. It is imperative to underline that a substantial number of bilateral trade observations are zeros, reflecting the need to use an appropriate estimator for this type of data.

**Table 3:** Descriptive statistics of the data. Source: Own elaboration

Variable	Mean	St. Dev	Min.	Max.
X_*Colj*_	174	763	0	9790
LogDIST_*Colj*_	9843.69	4635.13	733.53	19,369.97
CONTIG_*Colj*_	0.04	0.19	0	1.00
COMLANG_*Colj*_	0.14	0.35	0	1.00
LogGDP_*j*_	510,000	1,640,000	1240	17,900,000
LogLABCOMP_*Col*_	13,461.85	864.9	11,670.21	14,559.61
WTO_*j*_	0.86	0.34	0	1.00
OECD_*j*_	0.24	0.43	0	1.00
FTA_*ij*_	0.16	0.37	0	1.00
Control of corruption_*Col*_	− 0.30	0.09	− 0.41	− 0.12
Control of corruption_*j*_	0.05	1.06	− 1.67	2.47
Government effectiveness_*Col*_	− 0.06	0.09	− 0.25	0.07
Government effectiveness_*j*_	0.16	0.99	− 2.09	2.44
Political stability_*Col*_	− 1.40	0.42	− 2.06	− 0.77
Political stability_*j*_	− 0.04	0.95	− 2.97	1.62
Regulatory quality_*Col*_	0.30	0.14	0.01	0.50
Regulatory quality_*j*_	0.15	1.02	− 2.53	2.26
Rule of law_*Col*_	− 0.38	0.10	− 0.62	− 0.26
Rule of law_*j*_	0.06	1.03	− 2.34	2.10
Voice and accountability_*Col*_	− 0.07	0.13	− 0.32	0.19
Voice and accountability_*j*_	− 0.001	1.02	− 2.31	1.74
Observations	1904			

Exports and GDP data in millions of USD

Given this, a first definition considers the influence of institutional quality in levels over Colombian bilateral exports with its main partners, and this influence is modelled in the following specification:

1 $$X_{{{\text{Col}}j\,t}} = \exp \left( {\alpha_{o} +\, \beta_{1} {\text{Institutions}}_{{{\text{Col}}\,t - 1}} +\, \beta_{2} {\text{LogDIST}}_{{{\text{Col}}j}} + \,\beta_{3} {\text{CONTIG}}_{{{\text{Col}}j}} +\, \beta_{4} {\text{COMLANG}}_{{{\text{Col}}j}} +\, \beta_{5} {\text{LogGDP}}_{j\,t} +\, \beta_{6} {\text{LogLABCOMP}}_{{{\text{Col}}\,t}} + \beta_{7} {\text{WTO}}_{j\,t} +\, \beta_{8} {\text{OECD}}_{j\,t} +\, \beta_{9} {\text{FTA}}_{{{\text{Col}}j\,t}} } \right)n_{{{\text{Col}}j\,t}}$$

A second definition considers the difference between levels of institutional quality in Colombia and the destination countries (Colombian partners) in order to analyse the Colombian export panorama. The institutional quality difference between countries is commonly called institutional distance, and it captures in a simple way how the quality of institutions in the exporting country are better (positive) or worse (negative) than those in the importing country (Álvarez et al. 2018). In this regard, the authors conclude that a relative difference in favour of (or against) the exporting country promotes (or restricts) its exports due to their reduced (or wider) institutional distance. Consequently, the following specification is proposed:

2 $$X_{{{\text{Col}}j\,t}} = \exp \left( {\alpha_{o} +\, \beta_{1} {\text{DifInstitutions}}_{{{\text{Col}}j\,t - 1}} +\, \beta_{2} {\text{LogDIST}}_{{{\text{Col}}j}} + \beta_{3} {\text{CONTIG}}_{{{\text{Col}}j}} + \beta_{4} {\text{COMLANG}}_{{{\text{Col}}j}} +\, \beta_{5} {\text{LogGDP}}_{jt} + \beta_{6} {\text{LogLABCOMP}}_{{{\text{Col}}\,t}} + \beta_{7} {\text{WTO}}_{j\,t} +\, \beta_{8} {\text{OECD}}_{j\,t} +\, \beta_{9} {\text{FTA}}_{{{\text{Col}}j\,t}} } \right)n_{{{\text{Col}}j\,t}}$$

Finally, we estimate our models in their multiplicative form through the Poisson pseudo-maximum likelihood (PPML) estimator. This estimator has been widely used in recent studies due to its consistent results (Egger and Nigai 2015). Silva and Teneyro (2006) strongly recommend the use of the PPML estimator in trade gravity models rather than the ordinary least square (OLS) because the former includes a difference in the size of the coefficients, which are therefore smaller and more suitable. Additionally, the PPML estimator addresses potential endogeneity and econometric drawbacks such as bias produced by heteroscedasticity, serial correlated error, and multicollinearity (Álvarez et al. 2018). Moreover, the estimator is able to include zero values of trade in the specification, which is an advantage in the presence of a large number of zeros (Francois and Manchin 2013).

## Results

In this section, we present the results for the specifications proposed in the previous section, showing the influence of the institutions on Colombian exports. It is important to note that we estimate the models by including institutional variables one by one in order to avoid correlation problems between them.

Table 4 offers the results of the estimations of Colombian exports based on the gravity Eq. (1), where governance indices are included in terms of levels in the origin country. The estimation shows that the distance factor is statistically significant in each specification at a confidence level of 99%. However, the so-called control variables included (contiguity and common language) are not statistically significant. Similarly, the GDP variable of the destination, which represents the size of the economy, has a positive impact on exports and is statistically significant at a confidence level of 99%. Furthermore, the labour competitiveness variable in Colombia (as origin country), which indicates the productivity level per worker, and, therefore, the requirements for productive factors and wages, has a prominent positive effect on Colombian exports, which suggests that an increase in this factor should significantly boost exports. Regarding the variables that illustrate the Colombian trade integration mechanisms, the regression shows that there is no statistical significance between Colombian export levels and whether the destination country belongs to the WTO or OECD. These results raise questions around Colombia's recent accession to this organization in 2020 (OCDE 2020) as an economic mechanism with a favourable effect on its exports. Additionally, and contrary to the predominant positive results from different studies, the FTA variable exhibits a negative effect on Colombian exports, which suggests that when Colombia shares a trade agreement with its partners, its exports decrease.

**Table 4:** The influence of Colombian institutions on its exports

Variables	Control of corruption _(t − 1)_	Government effectiveness _(t − 1)_	Political stability _(t − 1)_	Regulatory quality _(t − 1)_	Rule of law _(t − 1)_	Voice and accountability _(t − 1)_
Institutional index_*Col* (t − 1)_	− 1.142**	0.673**	0.226***	0.993***	0.942***	0.522**
	(0.528)	(0.288)	(0.072)	(0.230)	(0.351)	(0.203)
LogDIST_*Colj*_	− 1.312***	− 1.311***	− 1.317***	− 1.316***	− 1.314***	− 1.315***
	(0.285)	(0.285)	(0.285)	(0.284)	(0.285)	(0.285)
CONTIG_*Colj*_	0.201	0.197	0.217	0.217	0.200	0.210
	(0.406)	(0.407)	(0.406)	(0.406)	(0.406)	(0.407)
COMLANG_*Colj*_	0.545	0.544	0.548	0.552	0.543	0.544
	(0.372)	(0.372)	(0.372)	(0.372)	(0.372)	(0.372)
LogGDP_*j*_	0.786***	0.786***	0.786***	0.786***	0.787***	0.786***
	(0.087)	(0.087)	(0.087)	(0.087)	(0.087)	(0.087)
LogLABCOMP_*Col*_	2.533**	2.944**	2.617***	2.044**	2.586**	2.990***
	(1.071)	(1.149)	(0.878)	(0.882)	(1.202)	(1.010)
WTO_j_	− 0.444	− 0.441	− 0.454	− 0.453	− 0.443	− 0.449
	(0.698)	(0.698)	(0.698)	(0.697)	(0.698)	(0.698)
OECD_*j*_	0.280	0.275	0.289	0.294	0.272	0.281
	(0.540)	(0.542)	(0.544)	(0.544)	(0.542)	(0.544)
FTA_*Colj*_	− 0.292*	− 0.284*	− 0.306*	− 0.316*	− 0.281*	− 0.293*
	(0.163)	(0.166)	(0.167)	(0.166)	(0.163)	(0.168)
Constant	− 14.791	− 18.342*	− 14.889*	− 10.070	− 14.621	− 18.738**
	(9.800)	(10.549)	(8.145)	(8.443)	(11.379)	(9.328)
Observations	1869	1869	1869	1869	1869	1869
R-squared	0.889	0.889	0.888	0.894	0.895	0.887
Reset test	0.654	0.681	0.683	0.699	0.691	0.681

Robust standard errors in parentheses. These are based on robust standard errors that have been adjusted for clustering by country pair

^***^p < 0.01

^**^p < 0.05

^*^p < 0.1

Regarding the variables of interest, we understand that all institutional indices are statistically significant and the majority of their results show the expected sign. The estimations show that the most relevant connections between trade and institutions are the rule of law and regulatory quality variables. The results are in line with prominent studies that have identified the substantial influence of these variables in the promotion of international trade (Álvarez et al. 2018; Anderson and Marcouiller 2002; Yu et al. 2015). Similarly, voice and accountability, government effectiveness, and political stability are variables demonstrating a notable effect on increasing Colombian exports, albeit at lower levels, in the latter variable in particular. On the other hand, the control of corruption variable exhibits a negative effect on Colombian exports, suggesting that improvements in this area could hinder exports. This result contradicts most of the empirical studies, which identified this variable as an export driver.

Table 5 reveals the results of the regressions of Colombian exports based on the gravity Eq. (2), focussing on the institutional distance between Colombia and its main partners. As mentioned, institutional distance is calculated as the difference between the governance indicators in Colombia (exporting country) and those of the destination country, to compare the institutional qualities of Colombia and its partners. The regressions show similar results in terms of influence on exports and statistical significance as those shown in Table 4 for variables such as distance, contiguity, GDP and labour competitiveness, and membership of the WTO and OECD. However, in this model, the common language variable becomes statistically significant when the institutional distance variables feature the same. Similarly, in this model, the FTA variable is not statistically significant.

**Table 5:** The influence of the institutional distance between Colombia and importing countries on Colombian exports

	Dif. control of corruption _(t − 1)_	Dif. governm. effectiv. _(t − 1)_	Dif. Political stability _(t − 1)_	Dif. regula. quality _(t − 1)_	Dif. rule of law _(t − 1)_	Dif. voice and accounta. _(t − 1)_
Institutional index_*Col* (t − 1)_	− 0.278**	− 0.416***	− 0.039	0.295**	− 0.295**	− 0.123
	(0.127)	(0.141)	(0.144)	(0.131)	(0.137)	(0.220)
LogDIST_*Colj*_	− 1.226***	− 1.231***	− 1.295***	− 1.277***	− 1.236***	− 1.273***
	(0.249)	(0.237)	(0.279)	(0.243)	(0.240)	(0.262)
CONTIG_*Colj*_	0.369	0.396	0.214	0.272	0.362	0.189
	(0.427)	(0.380)	(0.415)	(0.382)	(0.418)	(0.411)
COMLANG_*Colj*_	0.693*	0.669*	0.557	0.597*	0.654*	0.576
	(0.355)	(0.344)	(0.369)	(0.340)	(0.349)	(0.371)
LogGDP_*j*_	0.766***	0.757***	0.786***	0.781***	0.758***	0.787***
	(0.093)	(0.088)	(0.088)	(0.088)	(0.095)	(0.091)
LogLABCOMP_*Col*_	2.850**	2.663**	3.376***	3.025**	2.760**	3.161**
	(1.255)	(1.264)	(1.234)	(1.269)	(1.273)	(1.270)
WTO_j_	− 0.553	− 0.568	− 0.454	− 0.563	− 0.535	− 0.499
	(0.664)	(0.620)	(0.710)	(0.655)	(0.653)	(0.746)
OECD_*j*_	− 0.125	− 0.301	0.228	− 0.206	− 0.201	0.070
	(0.529)	(0.526)	(0.542)	(0.528)	(0.510)	(0.450)
FTA_*Colj*_	− 0.191	− 0.104	− 0.247	− 0.129	− 0.125	− 0.211
	(0.151)	(0.136)	(0.176)	(0.138)	(0.143)	(0.171)
Constant	− 17.712	− 15.622	− 22.677**	− 19.101	− 16.563	− 20.750*
	(12.019)	(12.274)	(11.251)	(12.263)	(12.472)	(11.977)
Observations	1869	1869	1869	1869	1869	1869
R-squared	0.894	0.890	0.887	0.883	0.890	0.890
Reset test	0.380	0.264	0.644	0.310	0.215	0.561

Robust standard errors in parentheses. These are based on robust standard errors that have been adjusted for clustering by country pair

***p < 0.01

**p < 0.05

*p < 0.1

Regarding institutional distance variables, Table 5 shows that the majority of them are statistically significant (except in the difference between political stability and voice and accountability), and that their effect on Colombian exports is adverse. As mentioned, a negative result in regression suggests a restriction of exports from Colombia to its partners due to the institutional distance, and the negative effect of institutional distance on Colombian exports is in line with indications from Bilgin et al. (2018). They affirm that a greater difference between the quality levels of the institutions in origin and destination countries negatively affects bilateral trade flows. The greatest adverse effect is shown in the distance between the effectiveness of the government variables, which suggests that differences between the quality of public services, capacity of the public function, and its independence from political pressures found between Colombia and its partners negatively affect the former’s exports. Nonetheless, the adverse effect of institutional distance on Colombian exports is also exhibited in the control of corruption, regulatory quality, and the rule of law variables, albeit to a lesser extent.

## Discussion

The results obtained by the proposed specifications allow us to examine the influence of institutional quality (whether we consider them in levels or their differences) on Colombian exports.

Regarding the impact of governance indices in levels on Colombian exports, the variables regulatory quality and rule of law exhibit a greater influence than the other indices, and this influence has been recognised by numerous studies. These variables are related to market competition and legal certainty, correspondingly, and our results reveal that their impact on the country's exports is outstanding. These findings should encourage the creation of more efficient policies to improve performance against those indices and what they represent in terms of governance as an effective measure to boost Colombian exports. In this regard, our results are in line with those revealed by Soeng and Cuyvers (2018), through which they provide evidence that Cambodia’s exports are positively influenced by the same variables we used in our study. Their study confirms too that the rule of law is the most relevant institutional variable for promoting the growth of Cambodia’s exports. On the other hand, the control of the corruption variable exhibits an adverse effect on exports, which reflects an ambiguous perspective on the social roots of corruption in the country and its diverse and opposite economic effects. This finding could be the initial basis of an additional study to determine the reasons for the negative effect of the control of corruption indicator on Colombian exports, a finding which contradicts recent research suggesting control of corruption has a positive effect on the volume of countries’ exports.

The prominent positive effect, exposed in each regression, that the labour competitiveness variable could have on Colombian exports is supported by the findings of Álvarez et al. (2018), who maintain that the labour productivity of the exporter, through lower factor requirements and salaries, boosts bilateral trade. With this in mind, it is essential to note that the promotion of policies that enhance Colombian labour productivity could be a powerful strategy to improve its exports, and our results confirm this statement. Nevertheless, the OECD (2019) affirms that Colombian labour productivity is on the decline; business tax breaks do little to improve the country’s productivity; and comparatively, Colombian labour productivity is one-third of that achieved by the average OECD country, and even lower than the level reached by some Latin American countries. These statements suggest that Colombian labour productivity may hardly be a factor in promoting the short-term growth of exports, and consequently, the reduction of Colombia’s trade deficit. Moreover, our findings show that once an FTA comes into effect between Colombia and its partners, it negatively affects Colombian exports, which questions the effectiveness of the core strategy of the Colombian government to promote its exports and eventually balance its trade deficit. The findings of the negative influence of the FTAs over Colombian exports with its partners differ from those found by Ahcar Olmos (2018) in his empirical study on international trade between Colombia and the EU, and those established by Cárdenas and García (2005) in their study on the effect on trade of the signing of the FTA between Colombia and the US.

Concerning the institutional distance and their influence on Colombian exports, it can be observed that the difference between governances in Colombia and its partners negatively affects its exports, with most of the variables included. The results are in line with those findings that indicate countries with similar (or different) standards of governance trade more (or less) with each other (de Groot et al. 2005). Moreover, the findings of Liu et al. (2020) regarding the effects of institutional distance on trade flows between China and countries following the Belt and Road Initiative (BRI), exhibit inhibition of the bilateral trade flows, deriving from uncertainty and transaction costs related to institutional distance. Nonetheless, it is important to mention that at least half of Colombian exports go to countries whose governance indices are generally higher (the US, the EU, and even some Chinese governance indices) than its own (see Fig. 1), which to some extent throws into question the common findings on institutional distance and the negative effect on exports when the distance is wide. Nevertheless, our findings should concern Colombian policymakers seeking to improve the level of institutional quality as a means to expand national exports with its partners, as the regression results suggest.

Overall, we can state that institutional quality has a strong influence on Colombian exports, where the effects of the regulation quality and rule of law variables stand out above the others. In the same vein, the institutional distance between Colombia and its partners represents another factor that harms exports. Therefore, the strengthening of institutional quality should be seen by the Colombian authorities as a primary strategy to support the trade integration process, focused not only on the opening of new markets through trade agreements, but also on measures that improve the economic, legal, social, and political environment of business.

## Conclusion

In this study, we have worked with a panel of the bilateral trade flows between Colombia and 136 of its partners to determine the influence of the country's institutional quality on its exports. To achieve this, we proposed a gravity model and considered concepts from the new trade theory. We included control variables related to economic size, geographical distance, cultural proximity, trade integration mechanisms, and labour competitiveness in order to estimate their effects on Colombian exports. In particular, the effects of institutional indices on Colombian exports flows were considered in levels and the institutional distances between countries. Our findings confirm the hypothesis that institutional quality has a positive effect on Colombian trade for most of the indices considered in the paper. However, the effects of the control of corruption index on exports differs from findings in previous studies, which suggest this variable has a vital role in the expansion of trade.

We conclude that in terms of Colombian exports, the results demonstrate that rule of law and regulatory quality have the most substantial impact, which reveals the need to improve conditions related to legal certainty and market competition in the country. Moreover, the estimations exhibit the remarkable effect on exports of the labour competitiveness variable. Nonetheless, as mentioned, Colombian labour productivity is declining, and the measures implemented by the government to enhance this indicator are not having the expected effect. Furthermore, unlike previous findings regarding the impact of trade agreements on exports, our results reveal that having an FTA in force hurts Colombian exports. Furthermore, it is crucial to point out that the variables that are usually considered in gravity models such as physical distance, GDP, contiguity, language, and an FTA, among others, include limited or even zero margin for improvement. By contrast, developing countries such as Colombia have a considerable margin for improvement in their institutional indicators (see Fig. 3), which could be a fundamental driver boosting its exports.

On the other hand, institutional distance estimates reveal that the differences in institution quality between Colombia and its partners reduce its volume of exports. These results confirm the need to improve Colombian governance indices, not only as a policy capable of improving institutional quality, but also as a policy that reduces the institutional distance from its partners, thereby promoting exports by reducing these institutional differences. Our findings support previous studies where it is concluded that institutional difference affects trade flows, as is the case with Colombian exports.

Our study offers some policy suggestions for Colombia, where governance has been influenced by an internal armed conflict that has lasted for more than 50 years. One suggestion concerns the outstanding effect of the variable rule of law on Colombian exports and the objective to improve the country’s legal framework, aimed mainly at strengthening its judicial system, which provides the conditions for improving the compliance of contracts and protection of private property. These advances will also have an indirect effect on promoting private investment. Another policy suggestion focuses on the regulation quality variable, oriented towards the improvements of conditions for the promotion of the private sector through the reinforcement of policies and regulations that support its development. Overall, Colombian policymakers should focus their efforts on improving each of the Colombian institutional quality indices because of the individual effect of each index on the expansion of exports and, therefore, on higher economic growth.

In general, the results of this research support the idea that institutional quality promotes exports between nations. Our study confirms the hypothesis regarding the influence of institutional quality on exports, regardless of whether we consider its influence in levels or institutional distance between Colombia and its partners.

However, one limitation of our study is that we estimate using aggregate trade data and we do not provide sectoral economic results. Another is the non-inclusion of informal governance indices such as the trust variable, which could provide different perspectives on the risk of default presumed by exporters to be present in an environment of poor effectiveness among formal institutions. Further research should also identify effective policies or measures that improve the country’s institutional quality level as a factor that strengthens business development in the republic and eventually promotes the growth of exports to its partners. Finally, we consider that future studies could empirically examine the impact of COVID-19 on the institutional quality of developing countries and, consequently, its effect on their exports.

## Data Availability

The datasets used and/or analysed during the current study are available from the corresponding author on reasonable request.
